# Editorial: Application of neuroscience in information systems and software engineering

**DOI:** 10.3389/fnins.2024.1402603

**Published:** 2024-11-19

**Authors:** Rüdiger Pryss, Jan vom Brocke, Manfred Reichert, Enrico Rukzio, Winfried Schlee, Barbara Weber

**Affiliations:** ^1^Institute of Clinical Epidemiology and Biometry, University of Würzburg, Würzburg, Germany; ^2^Institute of Medical Data Science, University Hospital of Würzburg, Würzburg, Germany; ^3^Department of Information Systems, University of Liechtenstein, Vaduz, Liechtenstein; ^4^Department of Information Systems, University of Münster, Münster, Germany; ^5^European Research Center for Information Systems (ERCIS), Münster, Germany; ^6^Institute of Databases and Information Systems, Ulm University, Ulm, Germany; ^7^Institute for Media Research and Media Development, Ulm University, Ulm, Germany; ^8^Institute for Information and Process Management, Eastern Switzerland University of Applied Sciences, St. Gallen, Switzerland; ^9^Department of Psychiatry and Psychotherapy, Interdisciplinary Tinnitus Clinic, University of Regensburg, Regensburg, Germany; ^10^Institute of Computer Science, University of St. Gallen, St. Gallen, Switzerland

**Keywords:** neuroscience, information systems, software engineering, cognitive load, cognitive psychology, brain-computer interfaces, process model comprehension

## 1 Motivation and Research Topic in numbers

The integration of methodologies from cognitive psychology and neuroscience into the study of IT systems has seen a notable uptick in recent years. This surge is spurred by the escalating complexity of IT systems, which increasingly tax both novice and seasoned users alike (Ma et al., 2023; Leger et al., 2014). Consequently, insights into constructing IT systems with a reduced cognitive burden are gaining paramount importance.

A survey of the current literature reveals several discernible trends. Researchers are delving into the cognitive processes involved in programming (Peitek et al., 2021), exploring the perceptual dynamics of virtual reality environments (Rixen et al., 2023; Holzwarth et al., 2021), deciphering how individuals comprehend process models (Abbad-Andaloussi et al., 2023; Winter et al., 2023), and examining the general perception of information systems (vom Brocke et al., 2020; Brocke et al., 2013). Moreover, there exist established models for analyzing the acceptance of information systems (Dwivedi et al., 2011), predominantly within the domain of computer science. It is also anticipated that the integration of artificial intelligence (AI) across various spheres of life will catalyze heightened research into the cognitive burden or alleviation offered by AI-driven IT systems (Gandhi et al., 2023). In technical terms, eye tracking (Scherer et al., 2012), think aloud protocols (Abbad Andaloussi et al., 2021), EEG (Zhang et al.), and measuring electrical skin conductivity (Winter et al., 2020) are commonly observed methods within the context of this Research Topic. The pinnacle of this field is exemplified by brain-computer interfaces (BCIs) (Fiani et al., 2021; Proverbio et al.), which establish a direct connection between the brain and input/output devices. With the proliferation of digital devices such as smartwatches and wristbands, as well as advanced facial recognition services, neuroscience is increasingly finding its way into everyday working and private life. Many use cases have been identified, including using emotion-sensing devices to significantly reduce stress and supporting flow in people's work processes (Whelan et al., 2019). Research has also begun to investigate the design of so-called neuro-adaptive processes, which capture body data during process execution and adapt the process flow and human-computer interaction accordingly in real time (vom Brocke, 2022).

Against this backdrop, this topic aims to foster a neuroscientific perspective on the subject matter. A total of 76 authors were actively solicited to contribute, with 29 ultimately participating. Out of the 22 targeted submissions, 17 papers were received. Following a rigorous review process, 12 papers were regrettably declined, while 5 were accepted, yielding an acceptance rate of 29%. Moreover, the topic has amassed 12,200 visits to date, underscoring the continued relevance of its content.

Spanning a duration of 11 months in 2022, this Research Topic enjoyed the collaborative support of both computer scientists and neuroscientists on the editorial board. In the subsequent chapters, we provide a comprehensive overview of the 5 accepted papers, culminating in a summary and future outlook in this editorial.

## 2 The papers of the Research Topic

In this Research Topic, five papers were accepted. These papers bear the following titles:

- On the accuracy of code complexity metrics: A neuroscience-based guideline for improvement (Hao et al.)
- Event-related brain potential markers of visual and auditory perception: A useful tool for brain computer interface systems (Proverbio et al.)
- Using spontaneous eye blink-related brain activity to investigate cognitive load during mobile map-assisted navigation (Cheng et al.)
- Don't overthink it: The paradoxical nature of expertise for the detection of errors in conceptual business process models (Boutin et al.)
- EEG emotion recognition based on cross-frequency granger causality feature extraction and fusion in the left and right hemispheres (Zhang et al.)

The papers can all be categorized as we discussed in the introduction. The following three papers heavily rely on EEG and cognitive load, underscoring the relevance of EEG as a method and the examination of mental load, validating their significance in the context of this topic.

First, the authors of Hao et al. present findings from an experiment involving 27 programmers, revealing that traditional code complexity metrics often fail to accurately capture the perceived complexity of code. Utilizing EEG measurements of cognitive load as a reference, the study suggests that these metrics may inadequately assess the difficulty programmers experience in comprehending code, prompting recommendations for refining existing metrics and proposing guidelines for future research in this domain.

Second, the authors of Cheng et al. delve into blink-related brain potentials, revealing that the visualization of landmarks on mobile maps impacts navigators' cognitive load in virtual environments, synthesizing cognitive neuroscience, navigation information system design, and brain-computer interface fields. It suggests that a mobile map featuring a moderate number of landmarks (specifically, five landmarks) optimally supports spatial learning without overwhelming navigators' attentional resources. Additionally, the study indicates a cognitive load spillover effect between map viewing and navigation, highlighting the validity of blink-related potential analysis as a method for assessing cognitive load during navigation.

Third, the authors of Zhang et al. introduce an emotion recognition scheme based on multi-feature extraction and fusion from EEG signals, leveraging the asymmetry of the brain hemispheres and Granger causality (GC) relationships. By categorizing EEG signals into four groups based on hemisphere and frequency, and employing an adaptive thresholding method (ATD) for feature extraction, the proposed scheme effectively enhances emotion recognition performance, as demonstrated by improvements of 8.43% compared to single GC features and 5.36% compared to direct cascade fusion methods on the DEAP emotion dataset, emphasizing the importance of considering cross-frequency causality in EEG signal analysis for enhanced emotion recognition.

The remaining two papers deal with the mental load in comprehending process models (Boutin et al.) and the improvement of brain-computer interfaces (Proverbio et al.), highlighting the relevance of process model comprehension and brain-computer interfaces.

The authors of Boutin et al. explore expertise in conceptual modeling and its impact on business analysts' performance, particularly focusing on visual attention and error detection processes. Results reveal unexpected findings where experts did not demonstrate significantly better performance compared to novices, suggesting potential biases and over-complexification among experts in error detection tasks. Future research avenues include investigating domain expertise, exploring brain activity using neuroimaging techniques, and considering nuanced measures like scan paths to enhance our understanding of expertise in conceptual modeling.

Finally, the authors of Proverbio et al. aim to identify reliable electrophysiological markers associated with visual and auditory perception of basic semantic categories, providing signals recognizable by brain-computer interface (BCI) systems for patients or device users. By contrasting brain signals across various perceptual categories in the same participants, the study uncovers unprecedented markers for sensory modalities, aiding in future development of automated classification systems. While categorization based on statistical analyses and expert supervision seems superior to machine learning systems for discriminating living stimuli, it requires human supervision for site and latency selection, suggesting potential for optimizing AI systems in reconstructing mental representations related to different categories of stimuli.

## 3 Summary

Many of the initially identified topics have been well-represented in the selected papers. EEG's prominence in measuring cognitive load stands out, reflecting its widespread use for this purpose. The increasing integration of process models in various industries highlights their growing importance, as evidenced by the inclusion of a paper on this subject. Moreover, the rising interest in brain-computer interfaces, evident in developments like Neuralink (Fiani et al., 2021) and concepts such as the Metaverse (Lee et al., 2021), indicate the emergence of new research avenues. Overall, these trends affirm the significance of neuroscience in fields like information systems or software engineering, pointing toward promising opportunities for future exploration.
